# Adsorption of methylene blue and tetracycline onto biomass-based material prepared by sulfuric acid reflux†

**DOI:** 10.1039/c8ra05395b

**Published:** 2018-09-19

**Authors:** Md. Tariqul Islam, Ricardo Saenz-Arana, Cesar Hernandez, Thomas Guinto, Md Ariful Ahsan, Hoejin Kim, Yirong Lin, Bonifacio Alvarado-Tenorio, Juan C. Noveron

**Affiliations:** Department of Chemistry, University of Texas El Paso, 500 West University Avenue El Paso Texas 79968 USA jcnoveron@utep.edu mtislam@miners.utep.edu tariqul.lab@gmail.com; Nanosystems Engineering Research Center for Nanotechnology-Enabled Water Treatment USA; Department of Mechanical Engineering, University of Texas El Paso, 500 West University Avenue El Paso Texas 79968 USA; Instituto de Ciencias Biomédicas, Universidad Autónoma de Ciudad Juárez Ciudad Juárez Chihuahua Mexico

## Abstract

The adsorptive removal of environmental pollutants is an effective method for the treatment of contaminated water. Thus, the preparation of adsorbents from low-cost, readily available, and renewable resources has garnered immense attention in recent years. In this study, a facile one-step method for the preparation of a high-capacity adsorbent is demonstrated by refluxing pine cones in concentrated sulfuric acid. With sulfuric acid reflux, the pine cones undergone carbonization as well as functionalization with sulfonic acid groups. The adsorbent demonstrated high adsorption capacity for two emerging organic pollutants, methylene blue (MB) and tetracycline (TC). Different variables such as pH, temperature, contact time, and initial concentration of the pollutants were analyzed and showed that the adsorption capacity for MB increased in a basic pH and *vice versa* for TC. Also, the elevated temperature favored the adsorption for both MB and TC. The maximum adsorption capacity was found to be 1666.66, and 357.14 mg g^−1^ for MB and TC, respectively. In comparison to the pristine pine cone, the sulfuric acid treated pine cone demonstrated an extraordinary improvement in the adsorption capacity. The adsorption of MB and TC was performed from the tap water matrix and similar adsorption capacities were found. A packed glass column was also prepared to demonstrate the adsorption of MB from tap water under flow conditions.

## Abstract 2

Facile conversion of pine cones into a high-capacity adsorbent for the removal of methylene blue and tetracycline from water.

## Graphical abstract

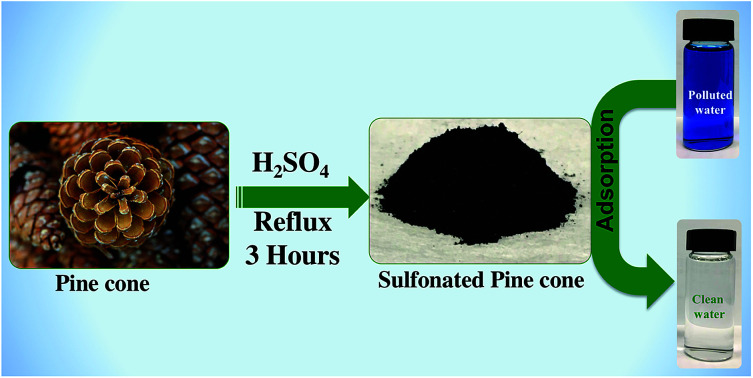

## 1. Introduction

Biomass conversion into fuels and commodity products has attracted considerable attention due to the large range of benefits of environmental sustainability.^1,2^ Although there has been an emphasis on the conversion of biomass to biofuels,^3^ the development of new methods to generate bio-derived products such as bio-plastics,^4^ bio-oils,^5^ biogas,^6^ bio-molecules,^7^ bio-char^8^ and most recently bio-carbon products has been increasing.^9^ Along the same lines, bio-derived adsorbents have attracted significant attention because they can provide an environmentally friendly and sustainable way to remove pollutants from wastewater.^10,11^ For example, modified and unmodified biomass derived adsorbents, such as dead biomass, living plants, fungi, bacteria, algae, yeast, waste sludge, agricultural wastes, biochar and activated carbon/charcoal, are widely utilized for the adsorption of organic and heavy metal pollutants from water.^12–15^ Because biomass is low-cost, highly abundant, and renewable; it has the advantage of being economically feasible to low-resource areas and thereby the biomass conversion into adsorbents has garnered immense attention in recent years.^16,17^ The utilization of biomass-derived adsorbents for the remediation of environmental pollutants from water can provide the access of clean water to rural and urban areas.

The rapid growth of population and increased industrialization are leading to the scarcity of water in many sectors.^18^ In the clean water sector, water contamination with organic compounds and heavy metals is becoming a serious health concern in both rural and urban areas. Therefore, clean water is becoming increasingly scarce almost everywhere in the world.^19^ For example, organic dyes are used in a variety of important industries, including but not limited to: textile, paper, paint, plastic, leather, food, cosmetic, and pharmaceuticals.^20^ In the textile industry, it is estimated that every year up to 20 000 tons of these dyes are lost in the process and end up in fresh water bodies.^21^ These untreated wastewaters synergistically pollute the usable freshwater supply. According to a report published by the US-EPA, many of these dyes are toxic and potential carcinogens to humans, which make their presence in the water a major health hazard.^22^ In addition to organic dyes, antibiotics have also become emerging water pollutants. Because of their improper disposal from hospitals, cattle and poultry industries; they are posing a new type of threat to the environment, as well as human health. The presence of antibiotics in the environment is not only unexpected, but also leads to the gradual increase in bacterial resistance. Therefore, the removal of antibiotics, like other pollutants, has become an essential part of wastewater treatment.^23^

Many different physical (adsorption, ultrafiltration, *etc.*),^24,25^ chemical catalytic (reduction, oxidation),^26–28^ photocatalytic,^29,30^ and electrocatalytic,^31^ and biological^32^ methods have been employed to remove or degrade the organic dyes and antibiotics. However, many of these methods are prohibitively costly, produce secondary chemical waste or are not effective enough to be applicable. In this regard, the adsorptive removal of pollutants from contaminated water has gained much attention, as the process is simple, less energy consuming, and does not cause secondary pollution.^33^ Commonly used adsorbents, such as activated carbon and ion exchange resins, are difficult to produce and thereby expensive in regards to the utilization in low-resource areas.^34^ Hence, the development of adsorbents from cheap and renewable sources, such as biomass, has become an intriguing alternative solution for the resource-limited areas.

Herein, we report a simple and scalable method for the preparation of a sulfonic acid functionalized carbonaceous adsorbent derived from pine cone for the removal of methylene blue and tetracycline from water. Pine cone is used because it is one of the widely available biomasses that has no such economical values. Additionally, as pine is a hard-wood type tree the fruit (pine cone) has a high percentage lignin.^35^ The lignin consists of aromatic rings and thereby can be simultaneously carbonized and functionalized to sulfonic acid group by the sulfuric acid reflux. The adsorbent was chemically and physically characterized by Fourier Transform Infrared Spectroscopy (FTIR), Thermogravimetric Analysis (TGA), Scanning Electron Microscopy (SEM), X-ray Powder Diffraction (XRPD), Energy Dispersive X-ray Scattering (EDS), X-ray Photoelectron Spectroscopy (XPS), and Raman spectroscopy. The FTIR, XRPD, EDS, XPS, and the Raman spectroscopy revealed the presence of sulfonic acid group and the carbonaceous nature whereas the SEM end TGA analyses demonstrated the morphology and thermal stability of the adsorbent, respectively. The sulfonated pinecone (PC-SO_3_H) was utilized for the adsorptive removal of MB and TC from water. The adsorption kinetics, isotherms, and thermodynamics were systematically studied and compared with the existing reports.

## 2. Materials and methods

### 2.1. Materials

Dry and mature pine cone was obtained from a local pine tree (El Paso, Texas) and was used without any further treatment. Concentrated sulfuric acid (95–98%), sodium hydroxide (>97%), were purchased from BDH Chemicals. Methylene blue (>98%) and tetracycline (≥98%) were purchased from Consolidated Chemical and Sigma Aldrich, respectively. Coarse sand was purchased from J. T. Baker (CAS number: 14808-60-7, MDL no. MFCD02100519). Ultrasonic bath sonicator (VWR 50T), having frequency of 60 Hz, was used to homogenize the adsorption mixture. Deionized water with a resistance of about 18.2 MΩ cm at 25 °C was obtained from Milli-Q water purifier facilities (EMD Millipore Corporation) from the lab. Tap water was obtained from the regular water faucet of the lab. The complete list of the soluble and insoluble species of the tap water is given in ESI, Fig. S1.†

### 2.2. Preparation of the adsorbent

**Scheme 1:**
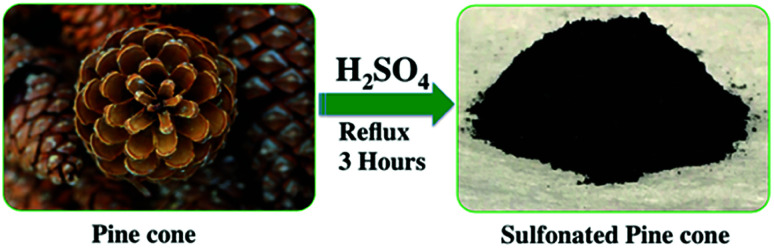
Preparation of sulfonated pine cone (PC-SO_3_H).

The preparation of sulfuric acid treated PC is depicted in Scheme 1. In detail, in a 300 mL round bottomed flask, 10 g of the unseeded and cut PC was mixed with 40 mL of concentrated H_2_SO_4_ (95–98%). An excess of H_2_SO_4_ was used to have a good suspension to ensure the reaction could be stirred properly during reflux. The reaction was refluxed in boiling H_2_SO_4_ for 3 h while stirring. After the reflux ended, the product mixture was cooled to room temperature, and 250 mL of cold water was slowly added to the mixture, with care so that the mixture didn't overheat or become volatile. Once the reflux finished, the reaction mixture was cooled down to room temperature and 250 mL of ice-cold water was slowly added to the mixture with due precautions.

Once the mixture was diluted, it was vacuum filtered and washed with about 3 L of DI water to remove the excess H_2_SO_4_. The sulfonated pine cone (PC-SO_3_H) was dried by placing it in a vacuum oven at 110 °C for 12 h. After the adsorbent had fully dried, it was ground to a fine powder with a ceramic mortar and pestle. The amount of PC-SO_3_H recovered was about 5.6 g.

### 2.3. Batch adsorption experiments

For the batch adsorption experiments of TC and MB, in a 25 mL glass vial, 20 mg of the PC-SO_3_H was added to a 20 mL solution of TC and MB, having initial concentrations varying from 300 to 2000 ppm for MB, and 50 to 500 ppm for TC. The mixture was then bath sonicated for 30 min, and then stirred at 1000 rpm on a magnetic stirrer for a period of 24 h. It was found that adsorption reached equilibrium after a period of 24 h and thereby a stirring period of 24 h was used for all the equilibrium adsorption experiments. After 24 h of stirring, 1 mL of the mixture was filtered through a 0.25 μm PTFE syringe filter to obtain a clear filtrate. The adsorption capacity was calculated from the difference between the initial and final concentration of MB and TC, which was obtained by using UV-vis spectrophotometer. The concentration of the TC and MB was obtained from their absorbance at 357 nm and 615 nm, respectively. The UV-vis spectrum and the calibration curves of MB and TC is given in Fig. S2.†

The pH-dependent adsorption of MB and TC was performed by dispersing 20 mg of the PC-SO_3_H in 20 mL of 1000 ppm MB and 200 ppm TC solutions, respectively. The pH of the solution was adjusted by using 1 M NaOH and 1 M HCl.

The time-dependent adsorption tests was obtained by adding 40 mg of the PC-SO_3_H in 40 mL of 500 ppm MB and 50 ppm TC solutions at pH ∼7 and ∼3.5, respectively. Acidic pH (∼3.5) for TC and neutral pH for MB was chosen as it was found that the adsorption of TC and MB was favored in those pH values. Bath sonication was carried out for 30 min and while sonication samples (∼0.5 mL) were taken at every 10 min interval. After the bath sonication, the mixture was stirred continuously on a magnetic stirring plate at 1000 rpm and during which samples were taken at every 30 min interval.

The temperature-dependent adsorptions were performed using 20 mg of the adsorbent in 20 mL of the MB and TC solutions at 23, 40, and 60 °C. Initial concentrations ranging from 300 to 600 ppm for MB and 200 to 400 ppm for TC were chosen for the adsorption of MB and TC. It was found that at 40 and 60 °C, TC undergone thermal degradation and thereby the absorbance at 357 nm dropped to some extent. Therefore, for the calculation of adsorption capacity at elevated temperatures, the amount of natural degradation was subtracted. However, no natural degradation of MB was found either at room temperature or at elevated temperatures.

### 2.4. Preparation of the adsorption column

**Scheme 2:**
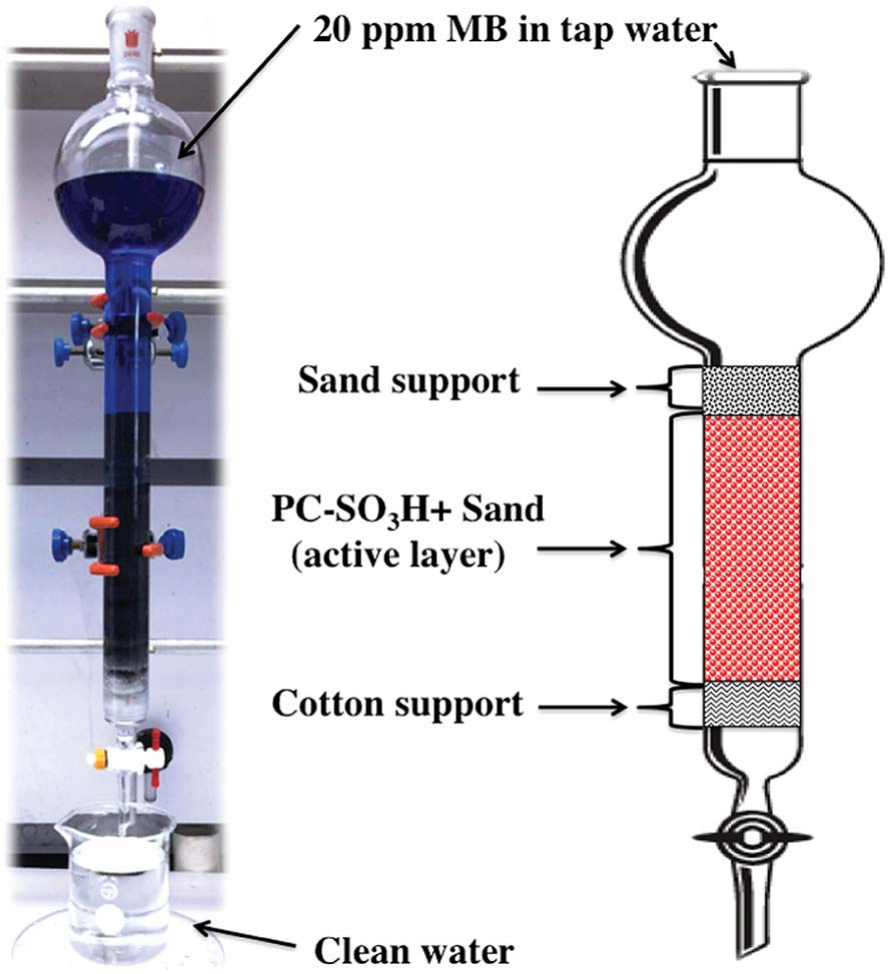
Design of the column filter packed with PC-SO_3_H and sand for the continuous adsorption of MB.

A fritted chromatographic column with reservoir having the dimensions of I.D. × *L* = 26 × 457 mm was packed to about 14 cm with the mixture of PC-SO_3_H and sand, Scheme 2. In detail, 2 g of the PC-SO_3_H was homogeneously mixed with 110 g of sea sand by a ceramic mortar and pestle for few minutes of gentle grinding. Afterwards, the column was filled by the dry filling method. To help disperse the flow as well as to prevent the leakage of any absorbent, cotton and coarse sea sand were used at the bottom and the top of the column, respectively.

The column was initially washed with 100 mL of DI water then a 20 ppm MB solution was prepared in tap water without the adjustments of the pH. However, it was found that the solution pH was about 7. The solution was fed into the column and allowed to flow through the filter under gravity. The flow rate varied from 6 to 6.8 mL min^−1^. For every 1000 mL of solution filtered, 2 mL of filtrate was sampled for UV-vis analysis. The filtration was continued until trace of MB was detected in the filtrate by naked eye observation.

### 2.5. Adsorption isotherms and kinetics

The experimental adsorption results were analyzed by utilizing the linear form of the Langmuir and Freundlich isotherm models, whereas time-dependent adsorption results were analyzed by utilizing the linear form of the pseudo-first and pseudo-second kinetic models. The model isotherms, such as the Langmuir and Freundlich isotherms, give important information about the adsorption processes. For example, the Langmuir isotherm is based on the monolayer type adsorption of molecules on the adsorbent. It is based on the assumption that the adsorption energy of each adsorbate molecule is the same and independent of the surface of the adsorbent. It also assumes that all sites on the adsorbent surface are equivalent and there are no interactions between the adsorbate molecules. The Freundlich model isotherm, on the other hand, is based the assumption that the adsorption of molecules happens both by the monolayer and multilayer on the adsorbent.

The linear form of the Langmuir and Freundlich adsorption isotherm models are expressed by the following equations.^36,37^1
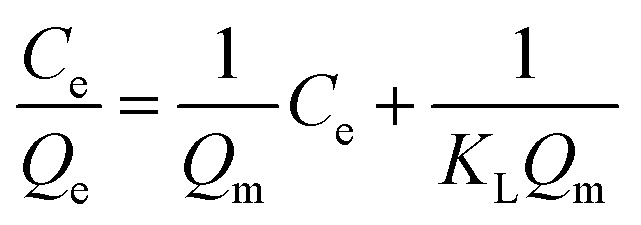
2
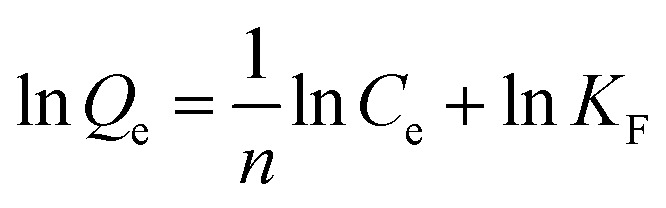
where *C*_e_ represents the equilibrium concentrations of the adsorbates (mg L^−1^), *Q*_e_ and *Q*_m_ represent the equilibrium and maximum adsorption capacities (mg g^−1^) of the adsorbent, *K*_L_ (L mg^−1^) is the Langmuir constant, which is related to the affinity of the binding sites and the free energy of adsorption.

*K*_F_ and *n* are Freundlich constants, determined from the intercept of the plot of ln(*Q*_e_) *versus* ln(*C*_e_). The *K*_F_ and 1/*n* are related to adsorption capacity and the adsorption intensity of the system. The magnitude of the term (1/*n*) indicates of the favorability of the adsorbent/adsorbate systems.

The linear form of the pseudo-second-order and the pseudo-first-order kinetic models are expressed by the following equation.^36,37^3
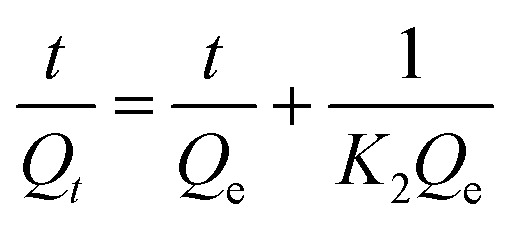
4
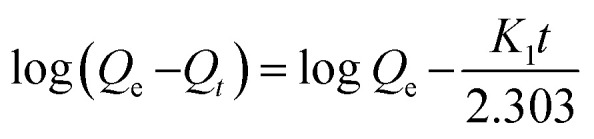
where *Q*_*t*_ represent the adsorption capacities (mg g^−1^) at time *t* (min); *K*_1_ (min^−1^) and *K*_2_ [g (mg^−1^ min^−1^)] are the pseudo-first-order and pseudo-second-order rate constants, respectively. The equilibrium and time-dependent adsorption capacities were calculated using the following equations:5
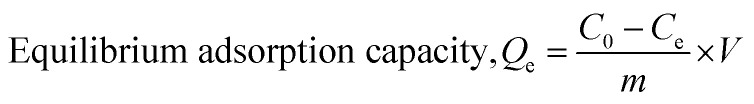


The % dye adsorption was calculated using the following equation:6
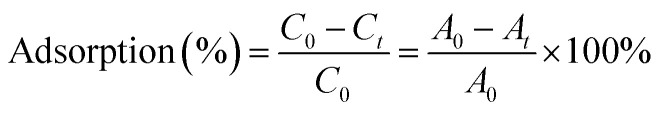
where *C*_0_, and *C*_*t*_ represent the concentrations of the dyes (mg L^−1^) at the beginning and at time *t*, respectively; *A*_0_ and *A*_*t*_ represent the absorbance of the dyes at concentrations *C*_0_ and *C*_*t*_, respectively; *m* represents the mass of the adsorbent (g) and *V* represents the volume of dye solution (L).

## 3. Results and discussion

### 3.1. Characterization of adsorbent

The PC-SO_3_H was chemically and physically characterized by Scanning Electron Microscopy (SEM) imaging, Energy Dispersive X-ray Spectroscopy (EDS), X-ray Photoelectron Spectroscopy (XPS), FTIR and Raman spectroscopy, X-ray Powder Diffraction (XRPD), Thermogravimetric Analysis (TGA), and zeta potential measurements. The morphology and the elemental composition of the PC-SO_3_H were characterized by the SEM and SEM-EDX analyses. X-ray Powder Diffraction analysis (XRPD) was used to determine the crystallinity and the carbonaceous nature of the PC-SO_3_H. FTIR and Raman spectroscopies gave further information about the functional groups and the type of carbon in the PC-SO_3_H. X-ray Photoelectron Spectroscopy (XPS) provided the insights about the elemental compositions as well as the type of bonding between different species in the PC-SO_3_H. The zeta potential analysis provided the net charge of the PC-SO_3_H in water, which eventually helped to understand the adsorption mechanism by electrostatic interactions. The N_2_ gas adsorption analysis (BET) was carried out to estimate the specific surface area of the PC-SO_3_H. It was found that the PC-SO_3_H had Brunauer–Emmett–Teller (BET) specific surface area of about 4.55 m^2^ g^−1^. In compared to the surface area of the modified and unmodified pine cone, reported in literature,^38^ the specific surface area of the PC-SO_3_H was found to be higher by several orders of magnitude. Along with the surface functionalization, the higher surface area of the PC-SO_3_H may be attributed to its exceptionally high adsorption capacity for MB and TC.

**Fig. 1:**
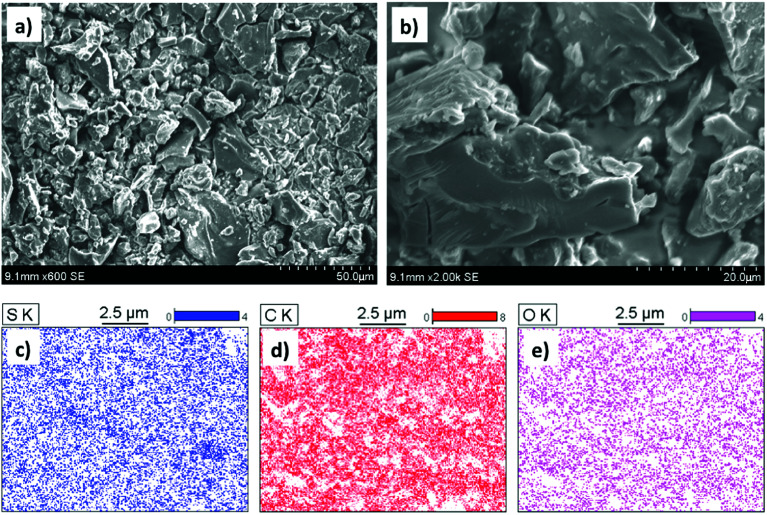
(a and b) SEM images of the PC-SO_3_H with varying magnifications and (c–e) elemental mapping images of the PC-SO_3_H depicting the presence of sulfur, carbon and the oxygen.

The surface morphology of the PC-SO_3_H was studied using scanning electron microscope (SEM). The SEM images revealed that the PC-SO_3_H consists of particles with random size and shape, Fig. 1a and b. The particles were found mostly in the micrometer size range with a large degree of aggregation. The surface of the PC-SO_3_H was seen rough and uneven without the presence of significant amount of pores.

**Fig. 2:**
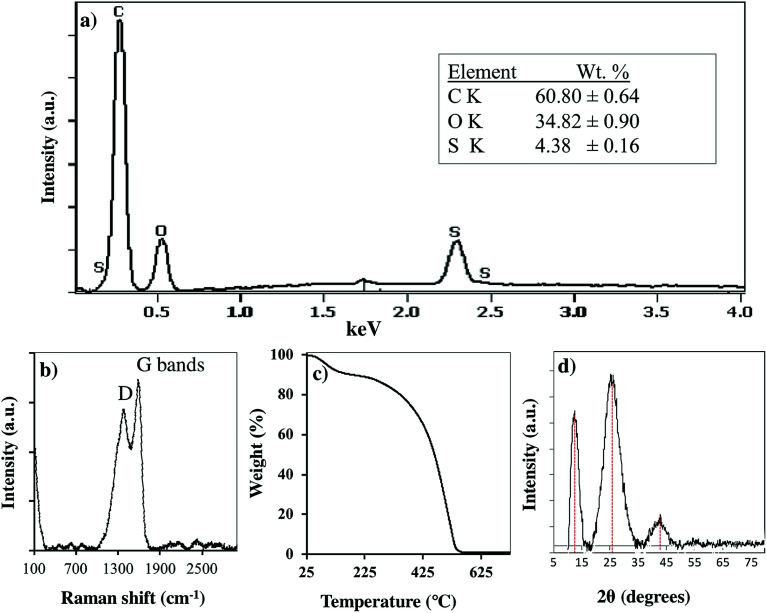
(a) EDS spectra showing the elemental composition, (b) Raman spectra shows the carbonaceous nature (c) the thermal gravimetric curve depicts the thermal stability and (d) XRPD pattern shows the crystalline carbonaceous nature of the PC-SO_3_H.

The SEM coupled with Energy Dispersive X-ray scattering (EDX) provided the qualitative and quantitative elemental analysis of the PC-SO_3_H. The EDX elemental mapping image demonstrated the presence and distribution of sulfur, carbon, and oxygen in the PC-SO_3_H, Fig. 1c and e. The presence even distribution of carbon, oxygen and sulfur further indicate the presence of –SO_3_H functional group in the adsorbent. The approximate qualitative and quantitative composition of the PC-SO_3_H was obtained from the EDS spectrum analysis, as shown in Fig. 2a. According to the EDX analysis, it was observed that the PC-SO_3_H was elementally composed of approximately 60.80% carbon, 34.82% oxygen, and 4.38% sulfur by weight.

Raman spectroscopy was used to examine the carbonaceous nature of the PC-SO_3_H. It also provided information about the degree of graphitization of the PC-SO_3_H and the imperfections in the carbon of the PC-SO_3_H, Fig. 2b. Two bands located at ∼1360 and 1582 cm^−1^ were observed, which are attributed to the D and G bands, respectively. The D band indicates to the structural defect and disorder and the G band corresponds to the sp^2^ carbon network in graphite-like carbon.^39,40^ In addition, the ratio of the intensities of the G to D band (*I*_D_/*I*_G_) is utilized to evaluate the extent of structural disorder for graphite-like carbon in the PC-SO_3_H. The *I*_D_/*I*_G_ ratio for the PC-SO_3_H was found to be 0.863, which indicated that the graphite-like carbon in the PC-SO_3_H has high degree of defects and disorder.^41^

As shown in Fig. 2d, the XRD pattern PC-SO_3_H has three major diffraction peaks at *ca.* 12.38, 25.73 and 42.25. The peaks at 25.38 and 42.25 could be indexed to the (002) and (101) planes of hexagonal graphite (JCPDS no. 41-1487), respectively.^42^ However, the peak at 12.38 could be indexed to the (002) plane of the stacked graphene oxide.^43^ Therefore, it could be considered that the PC-SO_3_H consists of graphitic as well as graphene oxide like carbons. According to the Bragg's equation (*nλ* = 2*d* sin *θ*), the inter layer spacing of the 002 and 101 planes of the graphitic carbon of the PC-SO_3_H was calculated as 3.475 Å and 2.137 Å, which are somewhat extended from the regular interlayer spacing of the graphite *viz*. *d*_002_ = 3.356 Å and *d*_101_ = 2.034 Å.^44^ The larger *d*_002_ and *d*_101_ values of the PC-SO_3_H compared to that of ideal graphite implied that the carbon is functionalized with different elements *viz.* oxygen and sulfur. Because of the functionalization the graphitic carbon in PC-SO_3_H is more extended and exfoliated.

The thermal stability and the decomposition profile of the PC-SO_3_H were determined by the Thermogravimetric Analysis (TGA). As shown in Fig. 2c, the PC-SO_3_H lost the free and absorbed water in the molecular space from a temperature range from 50 to 250 °C. The major decomposition of the adsorbent started from about 325 °C and a complete decomposition of the PC-SO_3_H was observed at a temperature of about 525 °C and above. Thus, the TGA curve indicated that the PC-SO_3_H has good thermal stability and is free from any metal oxide impurities.

**Fig. 3:**
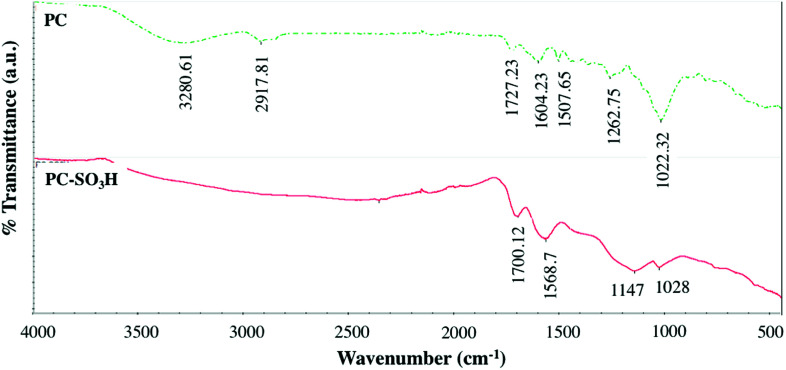
FTIR spectrum of the pine cone before and after the H_2_SO_4_ treatment.

Fourier transform infrared (FTIR) spectroscopic analysis was conducted on the pine cone before and after the sulfonation to identify the differences in the change in the functional groups of the PC-SO_3_H, Fig. 3. The FTIR spectra of the pine cone showed the presence of OH, aliphatic CH, carbonyl (C=O), and aromatic ring C=C functional groups by their characteristic stretching vibrations at 3280, 2919, 1727, and 1604 cm^−1^, respectively. The substituted aromatic ring and CH rocking vibrations could be located at 1472 and 1380 cm^−1^, respectively. The asymmetric bending vibration of CH_2_, the CO stretching of ether of cellulose, and the phenolic COH stretching could be assigned at 1430 cm^−1^, 1022 cm^−1^, and 1262 cm^−1^, respectively. In addition, two peaks at 1171 cm^−1^ and 1112 cm^−1^ could be assigned to the bending vibrations of CO. All these vibrations suggested the presence of alcohol, aromatic ring, methylene bridge and ether functional groups in the pristine pine cone.

After the sulfuric acid treatment, new peak originated at ∼1147 cm^−1^, which could be attributed to the –SO_3_H group in the PC-SO_3_H. Also, some characteristic peaks disappeared indicating the change in functional groups after the sulfuric acid reflux. For example, the OH peak of the pristine pine cone became almost flat, which indicated that the H_2_SO_4_ reflux caused the dehydration of the pine cone. The dehydration also converted the pine cone into a carbonaceous material. Therefore, from the Raman and FTIR spectroscopic analyses it could be concluded that the H_2_SO_4_ reflux caused the carbonization as well as sulfonation to the pine cone.

**Fig. 4:**
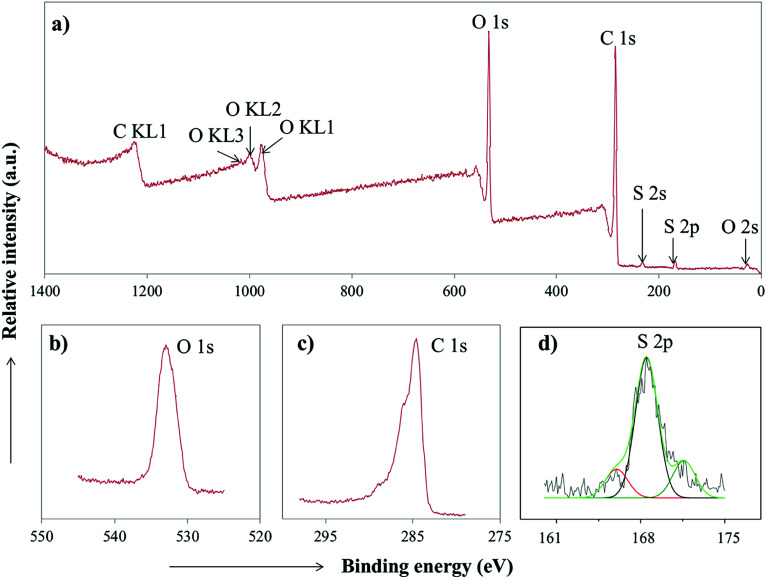
(a) XPS spectra of PC-SO_3_H. High-resolution XPS spectrum of (b) O 1s (c) C 1s and (d) S 2p of the PC-SO_3_H.

The composition of the PC-SO_3_H was further analyzed by high resolution X-ray Photoelectron Spectroscopy (XPS). The results revealed that PC-SO_3_H is elementally composed of carbon, oxygen and sulfur in which the sulfur is mainly associated with –SO_3_H functional group. Fig. 4d shows the S 2p3/2 and S 2p1/2 doublet with binding energies of about 169 eV, which could be assigned for the sulfonic acid functional group (–SO_3_H).^45–47^ An additional peak was identified at about 165 eV, which could be assigned to the S 2p1/2 binding energy of the thiol group^48^ that may have retained from the parent pine cone.

**Fig. 5:**
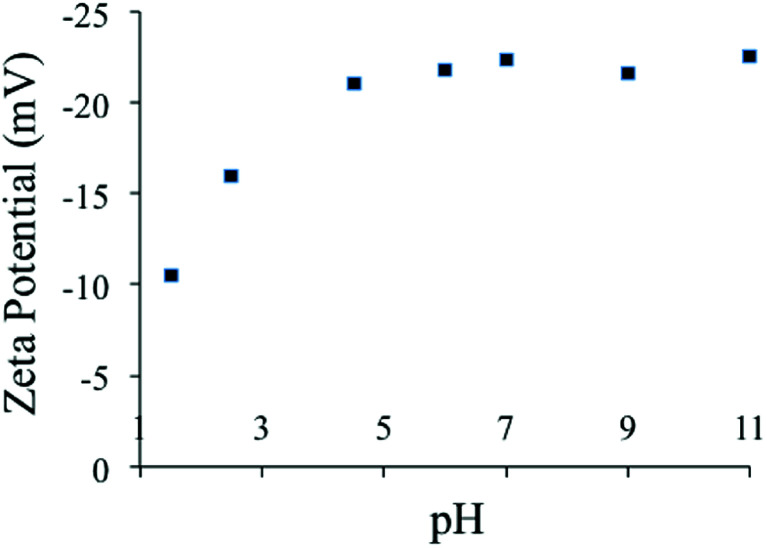
pH-dependent zeta potential of the PC-SO_3_H in water.

The zeta potential of the PC-SO_3_H was measured at the pH range of 1.5 to 11 in water, Fig. 5. It was found that the zeta potential of the PC-SO_3_H remains negative within the pH range. Due to the low p*K*_a_ of the sulfonic acid (R–SO_3_H, p*K*_a_ ∼ −7), the –SO_3_H group deprotonates to form sulfonate (PC-SO_3_^−^) and thereby the adsorbent demonstrated negative zeta potential under the experimental pH range.

It was observed that at pH 1.5 the negative zeta potential value is lowest and at pH above 4 the zeta potential remains almost similar. Therefore, at any pH within the range of 1.5 to 11, the PC-SO_3_H has the ability to adsorb the cationic pollutants by electrostatic as well as hydrophobic interactions.

### 3.2. Effect of pH on the adsorption of MB and TC

**Fig. 6:**
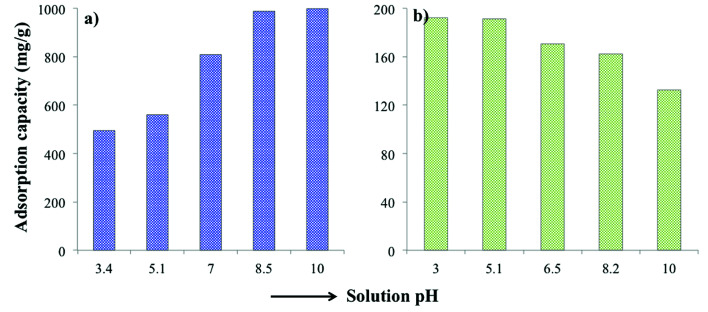
pH dependent adsorption of (a) MB and (b) TC by PC-SO_3_H, respectively. Initial MB concentration = 1000 mg L^−1^, *V* = 20 mL and adsorbent = 20 mg; initial TC concentration = 200 mg L^−1^, *V* = 20 mL and adsorbent = 20 mg.

The solution pH plays an important role in the adsorption efficiency. Therefore, several experiments were performed where the pH on MB and TC was varied from 3 to 10 in order to investigate the pH dependence of the adsorption. As MB is a basic dye, it stays positively charged when dissolved in water. In basic pH, PC-SO_3_H becomes more negatively charged, Fig. 5. Therefore, at basic pH the negatively charged adsorbent and the positively charged dye have higher electrostatic attraction. As a result, the adsorption capacity of PC-SO_3_H for MB increased at higher pH, Fig. 6a. In addition, MB can adsorb on the adsorbent by π–π interaction due to the conjugation in the MB structure the carbonaceous nature of the PC-SO_3_H. Similar results were obtained by other studies, where the adsorption of cationic dyes was favored at basic pH.^49^

On the other hand, the adsorption capacity for the TC onto PC-SO_3_H increased as the solution pH decreased, Fig. 6b. This response is assumed to cause by the protonation of the amine of the TC at acidic pH followed by the electrostatic interactions between the negatively charged sulfonate group of adsorbent and the ammonium group of the TC. Likewise MB, TC has conjugation in the structure and thereby it adsorbs on PC-SO3H by π–π interaction as well. These results correlate with previously published reports.^50^

### 3.3. Equilibrium adsorption capacity and the adsorption isotherms

**Fig. 7:**
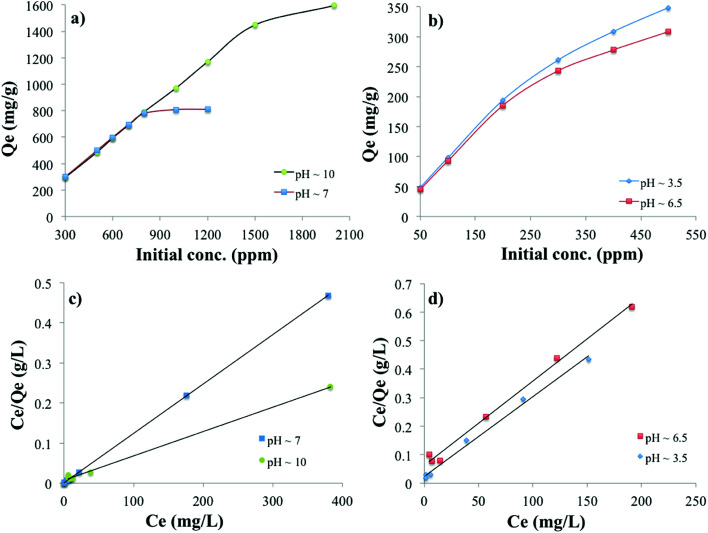
Adsorption capacity of PC-SO_3_H towards (a) MB and (b) TC at different initial pollutant concentrations and pH. Corresponding Langmuir isotherm models for the adsorption of (c) MB and (d) TC onto PC-SO_3_H.

The adsorption capacity of the PC-SO_3_H for MB and TC is shown in Fig. 7. It was found that the equilibrium adsorption capacity (*Q*_e_) for the MB adsorption increased with the increase in the solution pH and is *vice versa* for the adsorption of the TC. The adsorption capacity also increased with the increase in the initial concentration of MB and TC. The higher the initial concentration of adsorbate molecules, the stronger the driving forces to overcome the mass transfer resistances of adsorbate molecules from the aqueous to the solid phases, which results higher adsorption capacity. The data obtained from the equilibrium studies were analyzed according to Langmuir and Freundlich adsorption isotherm models. The *Q*_e_ was calculated by using the eqn (5). The adsorption capacities of PC-SO_3_H towards MB and TC with respect to the initial concentration and pH are shown in Fig. 7a and b. The *Q*_e_ was calculated to be about 1595 and 810 mg g^−1^ at initial MB concentration of 2000 and 1200 ppm and at pH 10 and 7, respectively (Fig. 7a). The MB adsorption capacity of PC-SO_3_H, found in this study, is much higher than the previously reported values, where pine cone derived adsorbent was prepared by other methods.^38,51^ For the adsorption of TC, the *Q*_e_ was measured to be 348 and 308 mg g^−1^ at the initial concentrations of 500 ppm, and the pH of 3.5 and 6.5, respectively (Fig. 7a). However, the pristine and unmodified pine cone showed adsorption capacity of 67 and 28 mg g^−1^ at pH ∼7 for MB and TC, Fig. S3.† In comparison to the pristine pine cone, the sulfuric acid treated pine cone demonstrated an extraordinary increase in the adsorption capacity for MB and TC. In order to avoid the confusion that the MB and TC may undergo natural degradation, control experiments were performed without the PC-SO_3_H. It was found that MB and TC did not degrade after 24 h of equilibration at room temperature (23 °C). Therefore, it could be inferred that the removal of these two pollutants from water happened by adsorption processes and sulfuric acid treatment did the extraordinary improvement in the adsorption capacity of the pine cone.

The linear form of the Langmuir isotherm model (eqn (1)), applied to the experimental data, is shown in Fig. 7c and d. A good fit of the experimental data with the Langmuir model isotherm suggested that the adsorption of MB and TC happened by monolayer type adsorption. Applying the Langmuir isotherm equation, the maximum adsorption capacity (*Q*_m_) was calculated to be 1666.66 and 833.33 mg g^−1^ for MB at pH 10 and 7, respectively. The *Q*_m_ for TC was calculated to be 357.14 and 333.33 mg g^−1^ at pH 3.5 and 6.5, respectively. The Freundlich adsorption isotherm model was also applied to the batch adsorption data for the MB and TC, Fig. S4.† The linear correlation coefficient (*R*^2^) of 0.896 and 0.740 were obtained for the MB adsorption at pH 10 and 7, respectively. The adsorption intensity (*n*), calculated from the isotherm plots, are 10.78 and 5.53 at pH 7 and 10, respectively. Since, the value of *n* is larger than 1, the adsorption of MB onto PC-SO_3_H could be suggested as a favorable process at both pH.^52^ The *R*^2^ values of the Freundlich isotherms for the adsorption of TC were found to be 0.843 and 0.838 at pH 3.5 and 6.5, respectively. The values of *n* were calculated to be 2.87 and 2.25 at pH 3.5 and 6.5, respectively. Therefore, the adsorption of TC onto the PC-SO_3_H could be suggested as a favorable process at both pH.

### 3.4. Adsorption kinetics

**Fig. 8:**
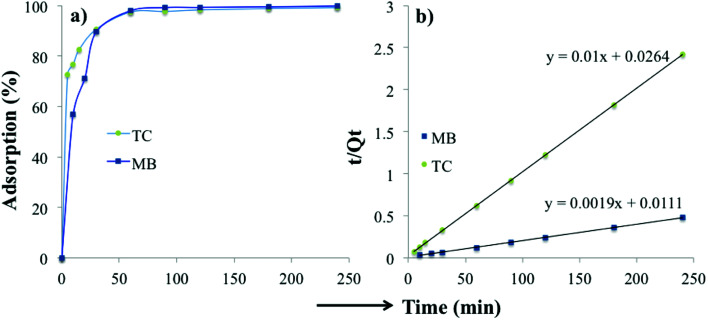
(a) Time-dependent percent adsorption of MB and TC and (b) the corresponding pseudo-second-order kinetics adsorption. Initial MB concentration = 200 mg L^−1^, *V* = 40 mL and adsorbent = 20 mg; initial TC concentration = 50 ppm, *V* = 20 mL and adsorbent = 10 mg.

The time-dependent adsorption of MB and TC by the PC-SO_3_H is shown in Fig. 8a. The results demonstrated a fast adsorption ability of the PC-SO_3_H and it was found that about 99% of the MB and TC was adsorbed within 100 minutes at pH 7 and 3.5, respectively. It could be observed that PC-SO_3_H has the fast MB and TC removal ability from the high concentration of MB and TC solutions *viz.* 200 and 50 ppm for MB and TC, respectively. The adsorption kinetics of MB and TC on PC-SO_3_H were investigated by two most commonly used kinetic models *viz.* pseudo-second-order and pseudo-first-order models. Fig. 8b shows the linear form of the Pseudo-second-order kinetic model, which was obtained by using eqn (3). For the adsorption of MB and TC, the relationship between the *t*/*Q*_*t*_*versus* time was found to be linear having the correlation coefficients (*R*^2^) values of 1. This suggested that the adsorption followed the pseudo-second-order kinetic model. The values of pseudo-second-order rate constants of MB adsorption were calculated to be 0.171 and 0.379 [g (mg^−1^ min^−1^)] for MB and TC, respectively.

However, the linear form of the pseudo-second-order kinetic model, applied on the experimental data by plotting log(*Q*_e_ − *Q*_*t*_) *versus* time (eqn (4)), gave a non-linear trend, Fig. S5.† The poor *R*^2^ values suggested that the pseudo-first-order kinetic model is inapplicable for the adsorption of MB and TC onto the PC-SO_3_H.

### 3.5. Adsorption thermodynamics

**Fig. 9:**
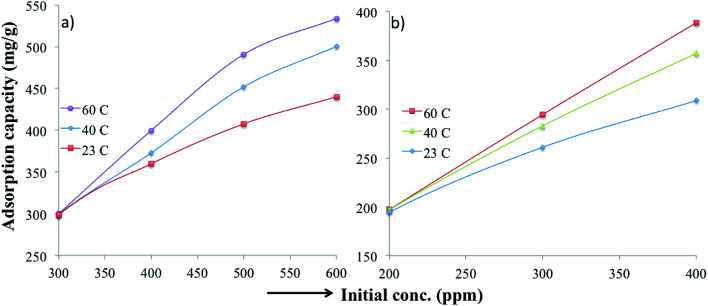
Temperature-dependent adsorption capacities of the PC-SO_3_H for (a) MB and (b) TC at pH 3.25 and 3.75, respectively.

Various thermodynamic parameters such as enthalpy, adsorption free energy and entropy changes were calculated for the adsorption of MB and TC over PC-SO_3_H. Fig. 9 depicts the temperature-dependent adsorption capacities at temperatures of 23, 40 and 60 °C. It was observed that the adsorption capacity for MB and TC increased with the experimental temperature, which indicated an endothermic adsorption process. At constant pH, the adsorption capacity for MB increased from 440 to 534 mg g^−1^ when the temperature was increased from 23 to 60 °C, Fig. 9a. For TC, the adsorption capacity increased from 309 to 388 mg g^−1^ with the same temperature increase, Fig. 9b. At elevated temperatures, the viscosity of the solution decreases and thereby the rate of diffusion of the adsorbate molecules across the external boundary layer and in the internal pores of the adsorbent particle increases.^53^ As a result, the adsorption capacity at elevated temperature increases. At elevated temperature, the adsorbent can be more hydrated, and the surface can become exposed to the adsorbate molecules to increase the adsorption capacity.

The Gibbs free energy change (Δ*G*) of adsorption was calculated using the following equation.^54^

7 Δ*G* = −*RT* ln(*K*_C_)

where *R* is the universal gas constant (8.31 J mol^−1^ K^−1^) and *K*_C_ is the thermodynamic equilibrium constant (L g^−1^), which can be obtained from the ratio of *Q*_e_ to *C*_e_.

The thermodynamic parameters for the adsorption MB and TC over PS-SO_3_H are shown in Table 1.

**Table 1:** Thermodynamic parameters for the adsorption MB and TC over PS-SO_3_H

Adsorbent	Adsorbates	Temperature (K)	*K*_C_ (L g^−1^)	Δ*G* (kJ mol^−1^)	Δ*H* (kJ mol^−1^)	Δ*S* (J mol^−1^ K^−1^)
PC-SO_3_H	MB	296	22.74	−76.83	23.82	106.67
		313	41.49	−96.90		
		333	66.78	−116.26		
	TC	296	66.16	−103.11	59.57	236.02
		313	239.51	−142.50		
		333	975.20	−190.45		

The Gibbs free energy change (Δ*G*) for the adsorption of MB at temperature 23, 40, and 60 °C was calculated to be −76.83, −96.90, and −116.26 kJ mol^−1^, respectively. The Δ*G* for the adsorption of TC at temperature 23, 40, and 60 °C was calculated to be −103.11, −142.50, and −190.45 kJ mol^−1^, respectively. The negative values of the Δ*G* suggested that the adsorption of MB and TC on the PC-SO_3_H was spontaneous process. The magnitude of the Δ*G* increased with increasing temperature indicating that the adsorption of MB and TC molecules is favorable at higher temperatures.

Additionally, the enthalpy and entropy changes (Δ*H* and Δ*S*) of adsorption were obtained using the van't Hoff equation.^55^8
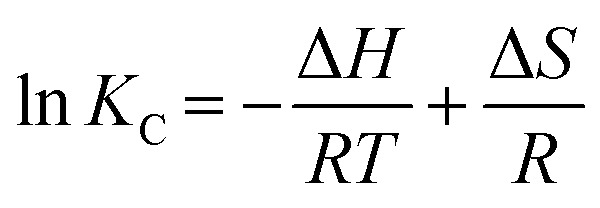


**Fig. 10:**
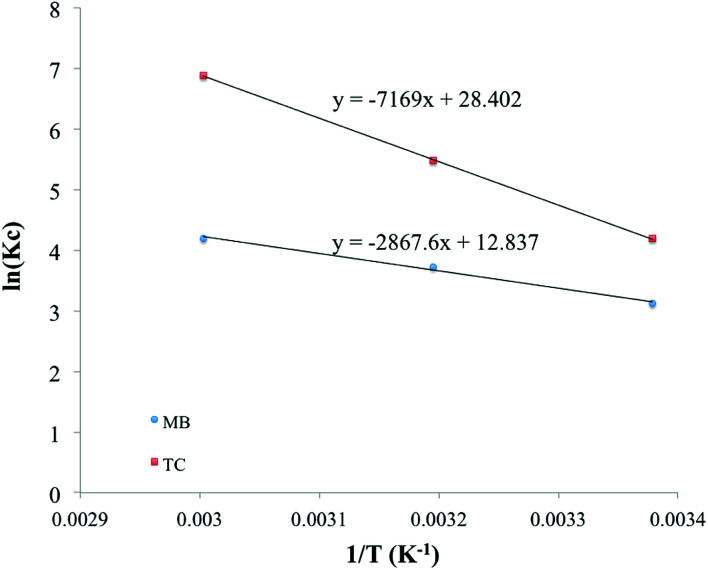
van't Hoff plots to obtain the Δ*H* and Δ*S* of the MB and TC adsorptions.

The van't Hoff graphs were obtained by plotting ln(*K*_C_) *vs.* 1/*T*.^56,57^ To determine *K*_C_, the ratio of *Q*_e_ to *C*_e_ was obtained from the adsorption experiments, where the initial MB concentration was 600 ppm. For TC, the initial concentration of 400 ppm was used to determine *K*_C_. The Δ*H* values, calculated from the van't Hoff plot (Fig. 10), were +23.82 and +59.87 kJ mol^−1^ for MB and TC, respectively. The positive values further indicate the endothermic adsorption process of MB and TC on the PS-SO_3_H. Moreover, as the Δ*H* values are less than 80 kJ mol^−1^, the adsorption process could be considered to happen by physisorption.

In addition, the values of the entropy changes (Δ*S*) were determined from the van't Hoff plot, Fig. 10. The Δ*S* values were calculated as +106.67, +236.02 J mol^−1^ K^−1^ for MB and TC, respectively. The positive values of the entropy changes indicated that the increase in the degrees of freedom or randomness at the adsorbent–adsorbate interface during the adsorption process. As Δ*S* is positive, applying the equation (Δ*G* = Δ*H* − *T*Δ*S*) it could be concluded that the spontaneity of the MB and TC adsorption is entropically favored.

As far our experimental parameters, the optimum conditions for the adsorption of MB was found at pH ∼10, temperature 60 °C, and after 24 h of equilibration time. However, for the adsorption of TC, the optimum condition was found at pH ∼3.5, temperature 60 °C, and after 24 h of equilibration time.

### 3.6. Adsorption of MB and TC from tap water

**Fig. 11:**
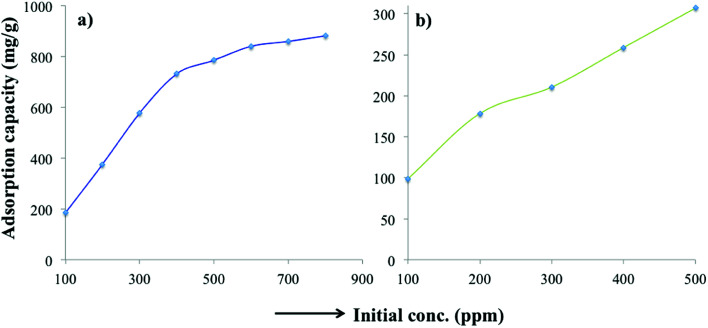
Adsorption capacity of PC-SO_3_H for (a) MB and (b) TC from tap water matrix. Initial MB concentration = 100–800 mg L^−1^, *V* = 20 mL and adsorbent = 10 mg; initial TC concentration = 100–500 mg L^−1^, *V* = 20 mL and adsorbent = 20 mg.

In addition to the organic pollutants (MB, TC), the wastewater usually contains many other soluble organic and inorganic species, which impede the adsorption efficiency. Therefore, the efficacy of the PC-SO_3_H was studied for the removal MB and TC of tap water as a realistic water matrix. The equilibrium adsorption capacity towards MB was found to be 880.6 at an initial MB concentration of 800 ppm and pH 7, Fig. 11. The equilibrium adsorption capacity towards TC was measured to be 306.8 mg g^−1^ at an initial TC concentration of 500 ppm and at pH about 7. From this study, it was found that the soluble ions present in the tap water had a minimal effect on the adsorption capacity. Therefore, it could be suggested that the PC-SO_3_H could potentially be utilized for the removal of MB and TC from drinking or tap water matrices.

### 3.7. Adsorbent packed column for continuous adsorption of MB

**Fig. 12:**
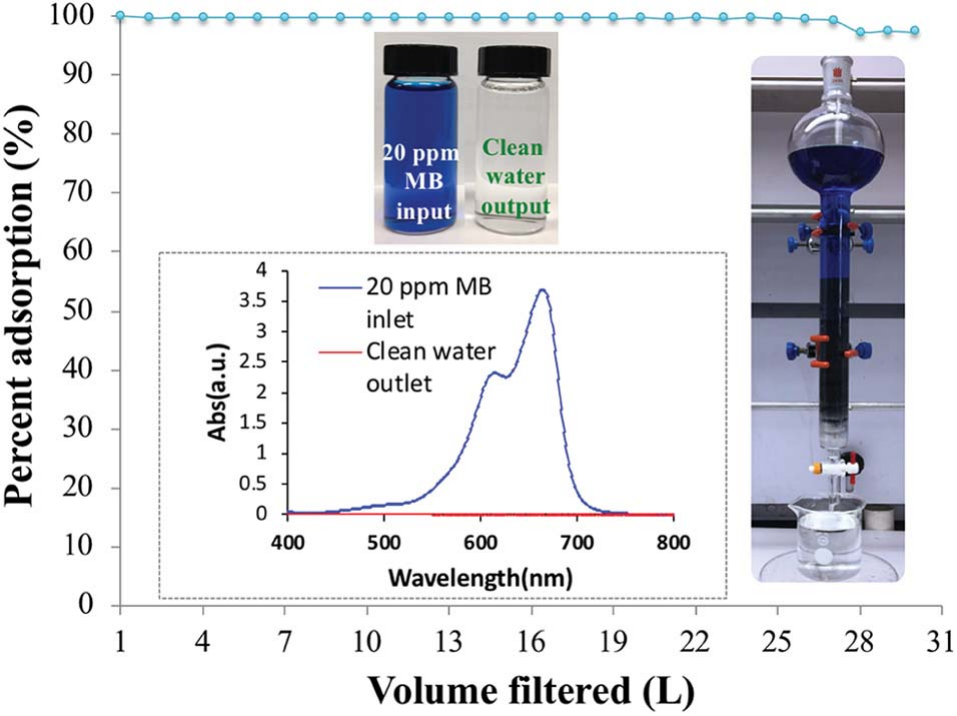
MB removal (%) *vs.* the volume of the 20 ppm MB solution filtered through the column. Insets: the feed MB solution and the clean filtrate; UV-vis spectrum of the 20 ppm MB solution and the clean filtrate; an operating filter.

To evaluate the dynamic behavior of MB removal by PC-SO_3_H, a continuous flow adsorption experiment was conducted by using a laboratory-scale packed glass column with the dimensions reported in the experimental section. The adsorption capacity of the PC-SO_3_H towards MB from tap water was examined under the continuous flow conditions through the packed column, Fig. 12.

The column filtered up to 27 liters of 20 ppm MB solution from tap water with 100% removal efficiency. After that, the column started to lose its efficiency and on the 30^th^ liter the removal efficiency dropped to ∼98%. The inset picture shows a functional packed column and the vials shows the 20 ppm MB solution inlet and the clean water outlet. As a control, a similar packed column was filled with sand and cotton support, without the PC-SO_3_H, and 20 ppm MB solution was filtered through it. It was found that the sand packed column could filter only about 0.5 L of water with 100% removal efficiency. Afterwards, the column drastically lost its efficiency and removed 90% MB at 0.75 L of water filtered. Therefore, from the continuous adsorption study through the column, it could be suggested that the PC-SO_3_H could be potentially utilized for the efficient removal of MB from the drinking or tap water matrices.

### 3.8. Comparison of the adsorption capacities with other adsorbents

Previous studies have investigated the adsorptive removal of MB and TC from aqueous solution utilizing low-cost based materials. The results of Table 2 illustrate the comparison of the adsorption capacities of different adsorbents under similar experimental conditions.

**Table 2:** Comparison of adsorption capacities of various adsorbents for the removal of MB and TC from deionized water

Adsorbent	Pollutants	*Q*_max_ (mg g^−1^)	*T* (K)	pH	References
Raw pine cone	MB	129	298	9	38
Base modified pine cone	MB	142	298	9	38
Sulfonated scrap tire	MB	833	296	10	58
Graphene	MB	153	293	7	59
Sulfonated tea waste	MB	1007	298	10	60
Bamboo charcoal	TC	23.5	303	7	61
Sulfonated scrap tire	TC	303	296	3.5	58
Carbon nanotubes	TC	269	298	2.3	62
Sulfonated tea waste	TC	381	298	4	60
Sulfonated saw dust	TC	270	298	4	63
PC-SO_3_H	MB	1667	298	10	This work
PC-SO_3_H	MB	833	298	7	This work
PC-SO_3_H	TC	357	298	3.5	This work
PC-SO_3_H	TC	333	298	6.5	This work

It shows that the PC-SO_3_H has better adsorption capacities for MB and TC compared to many other adsorbents such as raw or modified pine cone, activated charcoal, graphene, carbon nanotubes, sulfonated scrap tire and sulfonated spent tea leaf. Therefore, we believe that the PC-SO_3_H could be a potential candidate as the high-capacity and low-cost adsorbent for the removal of MB and TC in areas with insufficient resources.

### 3.9. Suggested adsorption mechanism

**Scheme 3:**
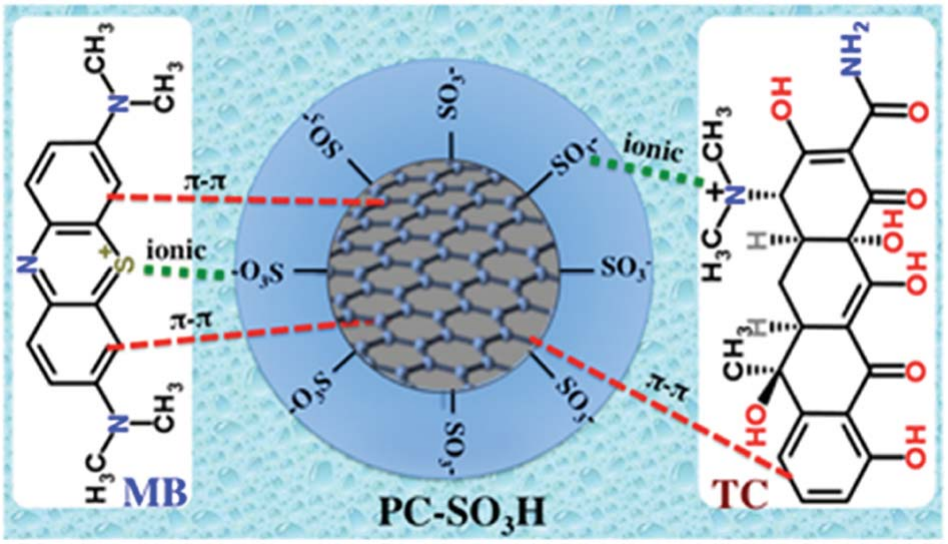
Proposed adsorption mechanism of the PC-SO_3_H towards the MB and TC.

Adsorption can happen by a number of different interactions *viz.* ionic or electrostatic interaction, π–π interaction, hydrogen bonding, van der Waals interaction, hydrophobic interaction, and so on. The chemical structure of MB and TC consists of aromatic ring with different degree of conjugations, which favors their adsorption on the carbonaceous adsorbent by the π–π stacking interaction, Scheme 3. Additionally, MB is a cationic dye and thereby it exists as positively charged in aqueous solution. From the zeta potential measurements, it was found that the PC-SO_3_H exists as negatively charged (PC-SO_3_^−^) throughout the pH range of 1.5 to 11, Fig. 5. Therefore, MB gets adsorbed on the PC-SO_3_H surface by the electrostatic attractive forces between the negative charge of the sulfonate group of the PC-SO_3_H and the positive charge of the MB.

On the other hand, TC has an aromatic ring with an extended conjugation system with various functional groups *viz.* phenol, alcohol, ketone, and tertiary amine and amide. At acidic pH < 3.3, TC becomes positively charged by the tertiary ammonium functional groups, whereas the PC-SO_3_H stays as PC-SO_3_^−^, Fig. 5. Therefore, at acidic pH the TC adsorption is favored by the ionic interaction between the sulfonate group of the adsorbent and the tertiary ammonium group of the TC. In the pH range from 3.3 to 7.68, the TC becomes mostly zwitterionic. Thus, the electrostatic interaction remains almost same within this range of pH and thereupon the adsorption capacity remains nearly identical. At basic pH > 8, the TC molecule becomes negatively charged by the phenolate and the hydroxylate functional groups,^64^ whereas the PC-SO_3_H exists as PC-SO_3_^−^, Fig. 5. Therefore, the electrostatic repulsive forces between the sulfonate group of the adsorbent and the negatively charged TC molecule lowers the adsorption capacity.

## 4. Conclusion

In conclusion, the preparation of a high-capacity and low-cost adsorbent based on pine cone is reported by a simple method of sulfuric acid treatment. As far our experimental conditions, the maximum adsorption of MB was found at pH ∼10, temperature 60 °C, and after 24 h of equilibration time. However, for the adsorption of TC, the maximum adsorption was found at pH ∼3.5, temperature 60 °C, and after 24 h of equilibration time. The maximum adsorption capacities were found to be 1666.66 mg g^−1^ for MB and 357.14 mg g^−1^ for TC. The pseudo-second-order kinetic model was in best fit with the experimental results. The adsorption of MB and TC followed the Langmuir isotherm model with better fit than the Freundlich model. A lab made packed column was prepared using the adsorbent, which filtered up to 27 L of 20 ppm MB solution from tap water with 100% removal efficiency. The sulfuric acid reflux method could be utilized for the preparation of high-capacity adsorbent from a wide variety of biomasses. The utilization of biomass to generate a high capacity and low-cost adsorbent can provide an environmentally friendly and sustainable way to remove anthropogenic pollutants from wastewater.

## Conflicts of interest

There are no conflicts exist.

## Supplementary Material

RA-008-C8RA05395B-s001
