# Supplementary material for: Adsorption of methylene blue and tetracycline onto biomass-based material prepared by sulfuric acid reflux
Source: RSC Adv. 2018 Sep 19;8(57):32545–57. doi: 10.1039/c8ra05395b (PMC9086250; doi:10.1039/c8ra05395b)
Supplement: RA-008-C8RA05395B-s001 [file RA-008-C8RA05395B-s001.pdf]

Electronic Supplementary Material (ESI) for RSC Advances.
This journal is © The Royal Society of Chemistry 2018

Supporting information

for

Adsorption of methylene blue and tetracycline onto biomass-based material prepared by sulfuric acid reflux

Md. Tariqul Islam,[ab*] Ricardo Saenz-Arana,[a] Cesar Hernandez,[a] Thomas Guinto, [a] Md Ariful Ahsan,[a] Hoejin Kim,[c] Yirong Lin,[c] Bonifacio Alvarado-Tenorio,[d] Juan C. Noveron[ab*]

a. Department of Chemistry, University of Texas, El Paso, 500 West University Avenue, El Paso, Texas 79968, USA.

b. Nanosystems Engineering Research Center for Nanotechnology-Enabled Water Treatment

c. Department of Mechanical Engineering, University of Texas, El Paso, 500 West University Avenue, El Paso, Texas 79968, USA.

c. Instituto de Ciencias Biomédicas, Universidad Autónoma de Ciudad Juárez, Ciudad Juárez, Chihuahua, México

Corresponding authors:

Juan C. Noveron, E-mail: jcnoveron@utep.edu

Md. Tariqul Islam, E-mail: mtislam@miners.utep.edu and tariqul.lab@gmail.com

Keywords: Pine cone, carbonization, sulfonation, adsorption, methylene blue, tetracycline, water treatment.

Characterization techniques

Hitachi S-3400N Type-II scanning electron microscope (SEM) equipped with an energy dispersive X-ray spectrometer with accelerating voltage of 15 kV was used to obtain SEM images and EDX spectrum. Copper tape was used as the substrate for both the imaging as well as the EDX analysis. Thermo ScientificTM Nicolet iS5 FTIR Spectrometer with attenuated total reflection (ATR) sampling technique was used for FTIR spectroscopy. UV-Vis spectrum was obtained by using Agilent Cary 50 Conc UV-Visible Spectrophotometer using quartz cuvette of volume 4 ml and path length 10 mm. Thermogravimetric analysis was performed by using a simultaneous DSC/TGA Q600 under nitrogen atmosphere, scanning at 10 °C/min from 25 °C up to 800 °C. Raman spectra were recorded on a micro-Raman LabRAM HR (Horiba Jobn Yvon, NJ, USA), equipped with a He-Ne laser (632.8 nm) was used as the excitation source. XPS data were collected using a Thermo Scientific Escalab 250Xi spectrometer with a six-channel detector. Photoelectrons were generated with a monochromatic Al Ka (1486.68 eV) X-ray source. Zeta potential measurements were carried out using a Zetasizer Nano ZS90 with a disposable folded capillary cell. For the zeta potential analysis, 10 mg of PC-SO_3H was suspended in deionized water and the pH of the mixture was adjusted by using 1M NaOH and 1 M HCl. Micromeritics ASAP 2020 Surface Area and Porosity Analyzer was used for nitrogen adsorption isotherms. Samples were weighed, heated at 150 °C, and evacuated at 10 mmHg for 12 h. After evacuation, nitrogen isotherms were obtained volumetrically at 77 K. The obtained data was fitted into the Brunauer- Emmett-Teller (BET) equation to determine the surface area.

DRINKING WATER ANALYSIS

Substance (Units)	Sample Year	Average Level	Minimum Level	Maximum Level	MCL	MCLG	Possible Source
**Turbidity**							
Turbidity (NTU)	2016	N/A	100%[1]	0.28	Treatment Technique	N/A	Soil runoff
**Inorganics**							
Antimony (ppb)	2016	0.09	0	0.3	6	6	Discharge from petroleum refineries, retardants, ceramics
Arsenic (ppb)	2016	4.9	0	10.7[2]	10	0	Erosion of natural deposits
Barium (ppm)	2016	0.09	0.04	0.18	2	2	Erosion of natural deposits
Chromium (ppb)	2016	2.82	1	6.60	100	100	Erosion of natural deposits
Selenium(ppb)	2016	1.90	0	5	50	50	Erosion of natural deposits
Fluoride (ppm)	2016	0.75	0.708	0.794	4	4	Erosion of natural deposits
Nitrate as Nitrogen (ppm)	2016	1.01	0.058	2.46	10	10	Runoff from fertilizer use
Gross Alpha (pCi/L)	2015	4.2	0	10.3	15	0	Erosion of natural deposits
Gross Beta (pCi/L)	2015	9.4	0	17.7	50	0	Decay of natural and man-made deposits
Combined Radium (pCi/L)	2015	0.09	0	1.2	5	0	Erosion of natural deposits
**Lead and Copper**							
Copper (ppm)	2016	0.12[3]	0.012	0.16	Action Level = 1.3	1.3	Corrosion of household plumbing systems
Lead (ppb)	2016	1.0[3]	0	8.0	Action Level = 15	0	Corrosion of household plumbing systems
**Coliform Bacteria**							
Total Coliform Bacteria	2016	N/A	0.0%	0.4%	5%	0	Naturally present in the environment
**Disinfection Residual**							
Chlorine (ppm)	2016	N/A[4]	N/A[4]	2.2	4[5]	4[6]	Water additive used to control microbes
Chlorine Dioxide (ppb)	2016	N/A[4]	N/A[4]	670	800[5]	800[6]	Water additive used to control microbes
**Disinfection Byproducts**							
Bromate (ppb)	2016	N/A[4]	N/A[4]	8.8	10	0	By-product of drinking water disinfection
Chlorite (ppm)	2016	N/A[4]	N/A[4]	0.316	1	0.8	By-product of drinking water disinfection
Total Haloacetic Acids (THAA) (ppb)	2016	12.3[8]	0	28.6	60	N/A	By-product of drinking water disinfection
Total Trihalomethanes (TTHM) (ppb)	2016	30.9[7]	0	66.5[9]	80	N/A	By-product of drinking water disinfection
**Total Organic Carbon**							
(Removal Ratio)	2016	2.49	2.08	3.02	Treatment Technique[10]	N/A	Naturally present in the environment
**Unregulated Contaminants[11]**							
Bromodichloromethane (ppb)	2016	10.52	0	21.7	N/A	0	By-product of drinking water disinfection
Chloroform (ppb)	2016	7.95	0	17.2	N/A	70	By-product of drinking water disinfection
Bromoform (ppb)	2016	4.70	0	11.8	N/A	0	By-product of drinking water disinfection
Dibromochloromethane (ppb)	2016	11.54	0	23.5	N/A	60	By-product of drinking water disinfection

(1) The lowest monthly percentage of samples meeting limits was 100%. Turbidity is monitored because it can interfere with disinfection.
(2) One sample measured 10.7 ppb; the running annual average for that location was 5.0 ppb.
(3) The lead and copper concentrations in 90 percent of the samples were at or below the levels shown. Samples are taken at the customer's tap.
(4) The average and minimum disinfection residuals are dependent on treatment techniques.
(5) Maximum Residual Disinfectant Level - The highest level of a disinfectant allowed in drinking water. There is convincing evidence that addition of a disinfectant is necessary for control of microbial contaminants.
(6) Maximum Residual Disinfectant Level Goal - The level of a drinking water disinfectant below which there is no known or expected risk to health. MRDLGs do not reflect the benefits of the use of disinfectants to control microbial contaminants.
(7) The highest running annual average at any location monitored was 58.5 ppb for TTHM.
(8) The highest running annual average at any location monitored was 22.9 ppb for THAA.
(9) One sample measured 66.5 ppb; the running annual average at that location was 58.5 ppb.
(10) The system is in compliance with a yearly removal ratio of 1.00 or greater.
(11) Unregulated contaminants are those for which EPA has not established drinking water standards. The purpose of unregulated contaminant monitoring is to assist EPA in determining the occurrence of unregulated contaminants in drinking water and whether future regulation is warranted.

Figure S1. List of different soluble species found in the tap water used for the experiments. Source: El Paso Water, 2016 Drinking water report. http://www.epwu.org/water/pdf/dwr_2016.pdf.

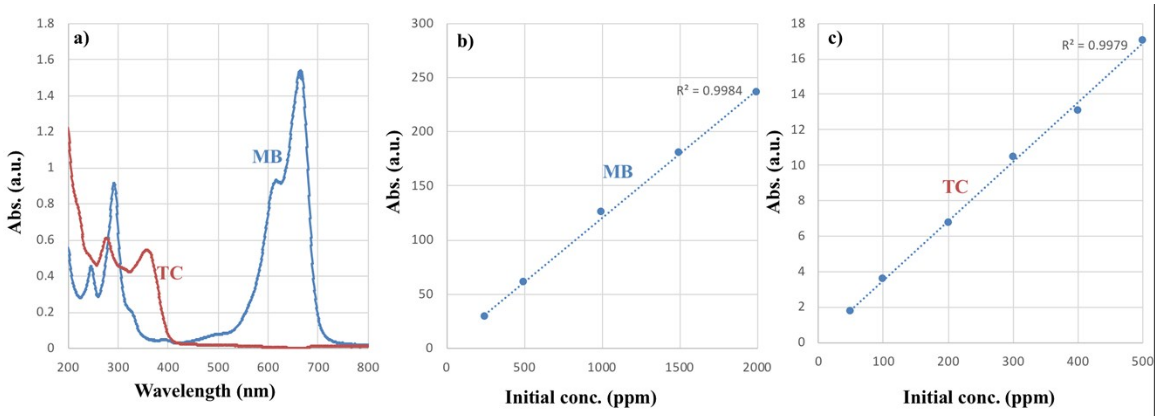

Figure S2. a) UV-visible spectrum of the MB and TC solution in water, b) calibration curve of MB, and c) calibration curve of TC.

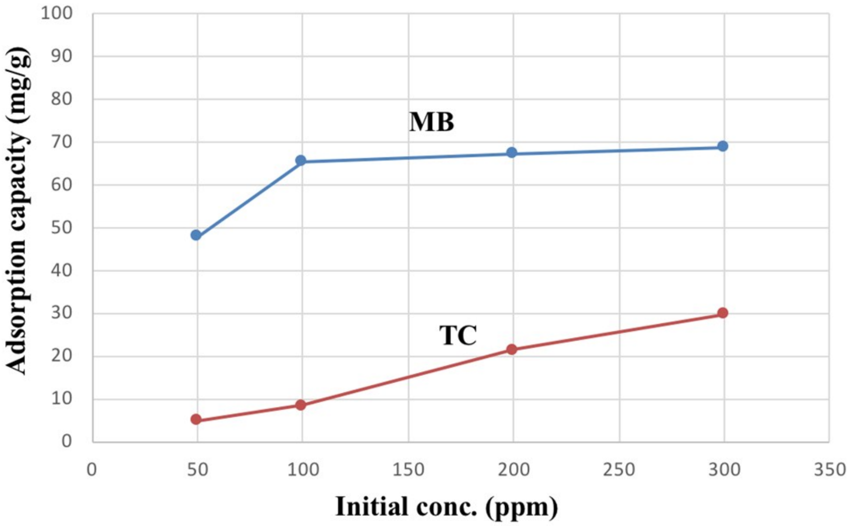

Figure S3. Adsorption capacity of the unmodified pine cone for MB and TC.

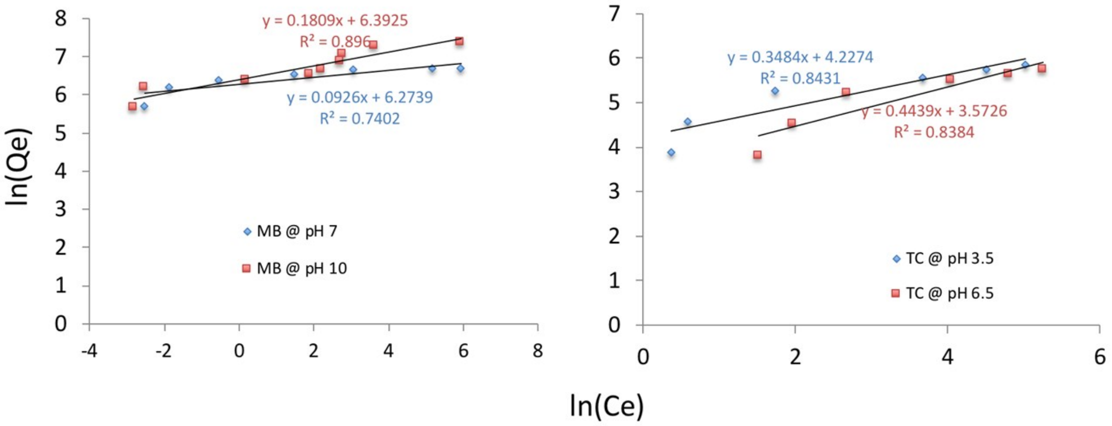

Figure S4. Freundlich isotherms for the adsorption of MB (left) and TC (right).

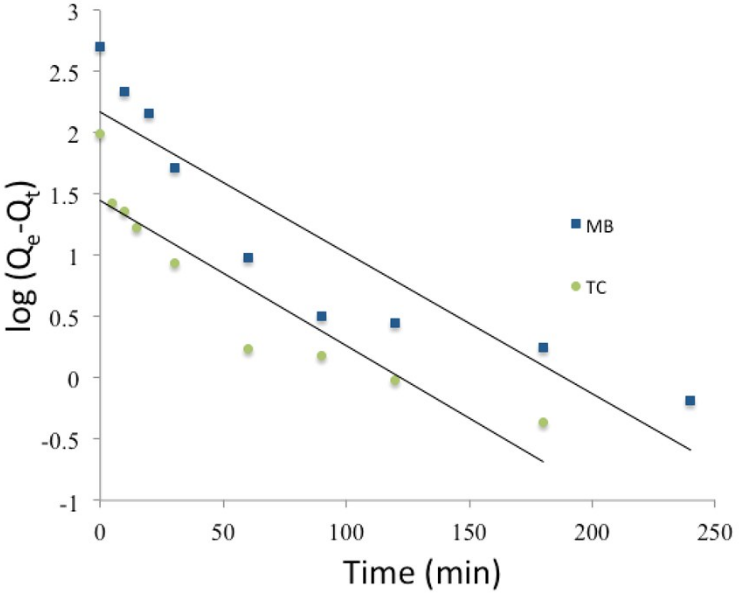

Figure S5. Pseudo-first order kinetics for the adsorption of MB and TC.
